# Effectiveness and Treatment Burden of Faricimab in Neovascular AMD: A Real-World Analysis of Treatment-Naïve and Previously Treated Patients in Poland

**DOI:** 10.3390/jcm15197602

**Published:** 2026-09-30

**Authors:** Michał Seweryn, Marek Rękas, Małgorzata Figurska

**Affiliations:** 1 Faculty of Health Science, Andrzej Frycz Modrzewski Krakow University, Gustawa Herlinga-Grudzińskiego 1, 30-705 Krakow, Poland; 2 Department of Ophthalmology, Military Institute of Medicine–National Research Institute, 04-141 Warsaw, Poland

**Keywords:** faricimab, neovascular age-related macular degeneration, anti-VEGF, real-world evidence, treatment burden, injection interval, visual acuity, registry study, Poland

## Abstract

**Background/Objectives:** Faricimab is a bispecific antibody targeting vascular endothelial growth factor A and angiopoietin-2, developed to extend treatment intervals in neovascular age-related macular degeneration (nAMD). Real-world evidence on faricimab within the Polish national drug program remains scarce. This study aimed to assess one-year clinical and treatment outcomes of faricimab for nAMD using real-world data from the Polish Therapeutic Program Monitoring System (SMPT). **Methods:** In this retrospective, non-randomized registry study, 322 patient-eye records treated with faricimab within the national drug program between January 2024 and April 2025 were analysed. Eligibility required at least 360 days between the first and last injections. Eyes were stratified into treatment-naïve (*n* = 70) and continuation (switched from another anti-vascular endothelial growth factor (anti-VEGF) agent; *n* = 252). **Results:** Median baseline best-corrected visual acuity (BCVA) was 70 [60, 77] letters overall, with no significant difference between subgroups (*p* = 0.59). At one year, treatment-naïve eyes improved to 75 [66, 79] letters (Δ +5 [0, 10]), whereas continuation eyes were maintained (Δ 0 [−5, 5]; between-group *p* < 0.001). A clinically meaningful (≥10-letter) gain occurred in 16% of eyes overall, more often in treatment-naïve than continuation eyes (27% vs. 12%; *p* = 0.004). Central retinal thickness (CRT) decreased significantly in all subgroups, with a greater reduction in treatment-naïve eyes. Intraocular pressure (IOP) remained stable (median Δ 0 mmHg). Patients received a median of 8 [7, 8] injections; injection numbers fell from 5 [4, 5] in the first half-year to 3 [2, 3] in the second, and intervals extended from approximately four to eight weeks. **Conclusions:** Within the Polish program, one year of faricimab was associated with stable-to-improved vision, anatomical improvement, and progressive extension of injection intervals after the mandated loading phase. Given the observational, single-arm design and the persister-only cohort, findings represent associations rather than comparative effectiveness.

## 1. Introduction

Neovascular age-related macular degeneration (nAMD) is one of the leading causes of irreversible visual impairment among older adults worldwide. The global prevalence of age-related macular degeneration is projected to reach approximately 288 million individuals by 2040, driven primarily by population aging and increasing life expectancy. Visual loss associated with nAMD significantly affects quality of life and daily functioning, particularly activities requiring central vision such as reading, driving, and face recognition [1].

The introduction of intravitreal anti-vascular endothelial growth factor (anti-VEGF) therapy has transformed the treatment of nAMD. Agents such as ranibizumab and aflibercept have demonstrated substantial efficacy in large randomized clinical trials, significantly reducing the risk of vision loss and enabling visual acuity improvement in a considerable proportion of patients [2,3].

More recently, therapeutic strategies have shifted toward longer-acting agents and individualized regimens designed to reduce treatment burden while preserving efficacy. Faricimab is a bispecific antibody that simultaneously targets vascular endothelial growth factor A (VEGF-A) and angiopoietin-2 (Ang-2), thereby addressing two complementary pathways involved in retinal vascular instability and inflammation. In the phase 3 TENAYA and LUCERNE trials, faricimab given at extended intervals of up to 16 weeks achieved BCVA outcomes non-inferior to aflibercept every 8 weeks, with approximately 45% of patients maintained on 16-week dosing at one year [4]. The therapeutic landscape has since broadened with other durable regimens: high-dose aflibercept 8 mg achieved visual gains non-inferior to aflibercept 2 mg while permitting dosing intervals of up to 16 weeks in the phase 3 PULSAR trial [5], and brolucizumab enabled 12-week dosing in a large proportion of eyes in the HAWK and HARRIER trials, albeit with a recognised risk of intraocular inflammation [6].

In Poland, anti-VEGF therapy for nAMD is delivered through a public drug program and monitored in the Therapeutic Program Monitoring System (SMPT). Registry-based analyses of this system have provided valuable real-world evidence on the effectiveness of anti-VEGF therapy [7], while population-level analyses highlight growing demand for retinal disease treatment and the importance of optimizing access to therapy within the national program [8]. Real-world data on faricimab specifically within the Polish national drug program, however, remain scarce. As a benchmark, a recent analysis of the same national registry reported that, across anti-VEGF agents, only approximately 22% of eyes achieved a clinically relevant one-year visual gain (≥0.3 logMAR, ≈15 ETDRS letters) and that patients received a mean of approximately six to seven injections per year [7], underscoring the gap between trial efficacy and routine-care effectiveness against which faricimab should be evaluated.

Therefore, the aim of the present study was to assess one-year clinical and treatment outcomes of faricimab therapy for neovascular age-related macular degeneration using real-world data from the Polish Therapeutic Program Monitoring System (SMPT). In particular, we analyzed functional and anatomical outcomes, treatment intensity, and injection intervals in treatment-naïve patients and in patients previously treated with other anti-VEGF agents.

## 2. Materials and Methods

This was a retrospective, non-randomized observational study based on real-world data from the Polish Therapeutic Program Monitoring System (SMPT). The analysis covered the period from January 2024 to April 2025 and included 322 patient-eye records with nAMD treated with faricimab. The extract contained one treated-eye record per patient identifier. Laterality and second-eye linkage were not available; therefore, the possibility that bilateral treatment could introduce residual non-independence could not be fully assessed. A minimum treatment duration of 360 days—defined as the interval between the first and the last intravitreal faricimab injections within the therapeutic program—was required to ensure a clinically meaningful follow-up of at least one year and to allow assessment of one-year functional, anatomical, and treatment-interval outcomes. Under the B.70 drug programme, faricimab is administered as a monthly loading phase (injections every 28–35 days; four loading doses during the study period [9], reduced to three from April 2025 [10]), followed by a treat-and-extend maintenance phase with a minimum interval of 8 weeks and a maximum of 16 weeks [9]. Patients were stratified into two subgroups: treatment-naïve (no prior anti-VEGF therapy before starting faricimab) and continuation patients (previously treated with another anti-VEGF agent before switching to faricimab). Outcomes were assessed at injection-anchored time points: baseline (first faricimab injection), first half-year (last injection on or before day 183), second half-year (last injection after day 183 within the analysed first-year course), and one year (the last injection of the analysed first-year course). As eligibility required at least 360 days between the first and last injections, the one-year assessment occurred at a median of 378 days (IQR 371–392; range 361–462) after initiation. The analyzed parameters included best-corrected visual acuity (BCVA) expressed in ETDRS letters, intraocular pressure (IOP, mmHg), and central retinal thickness (CRT, µm). BCVA values recorded in Snellen charts in SMPT were converted to ETDRS letter scores according to the method of Gregori et al. [11]. Categorical visual outcomes at one year were classified using standard ETDRS strata (loss ≥ 15, loss 5–14, stable within ±4, gain 5–14, and gain ≥ 15 letters); a clinically meaningful change was additionally defined as a gain or loss of ≥10 ETDRS letters.

Analyses were performed both within subgroups and between treatment-naïve and continuation patients. Within-group comparisons addressed the number of injections (first vs. second half-year); changes in BCVA, IOP, and CRT at different time points; injection intervals; and BCVA dynamics relative to the number of injections. Between-group comparisons included the number of injections across periods, baseline and subsequent changes (Δ) in clinical parameters, injection intervals, and the proportion of patients with clinically meaningful BCVA improvement or deterioration. When significant baseline differences in a parameter were observed (e.g., CRT), between-group inference for that parameter was based on the change from baseline (Δ). Absolute between-group values at follow-up are presented in the Results for completeness but, given the baseline imbalance, are reported for descriptive purposes only and were not used for formal between-group inference.

Statistical analysis was performed in R version 4.6.0 (R Foundation for Statistical Computing, Vienna, Austria) using RStudio version 2026.05.0+218 (Posit Software, PBC, Boston, MA, USA), following preliminary data processing in Power BI (Microsoft Corporation, Redmond, WA, USA). The Shapiro–Wilk test was used to assess normality of distribution. Due to non-normal distribution of variables, results are presented as medians with interquartile ranges (IQRs). Comparisons between independent groups were made using the Mann–Whitney U test, while paired data were analyzed with the Wilcoxon signed-rank test. Differences in proportions between subgroups were assessed with the Chi-square test. A *p*-value of <0.05 was considered statistically significant. Analyses were performed on available (complete-case) data at each time point, without imputation of missing values. Given the exploratory design and the large number of comparisons, *p*-values were not adjusted for multiplicity and are reported as descriptive measures. Emphasis was placed on effect sizes and on predefined thresholds of clinical relevance (a change of ≥5 and ≥10 ETDRS letters for BCVA) rather than on statistical significance alone. Statistically significant differences with a median magnitude below these thresholds—or, for IOP, without a clinically relevant change—were not regarded as clinically meaningful. Accordingly, isolated statistically significant findings should be interpreted cautiously. To account for baseline imbalances, between-group differences in the one-year change in BCVA and CRT were additionally assessed using analysis of covariance (ANCOVA) adjusted for the corresponding baseline value. The trajectory of BCVA across successive injections was modelled with a linear mixed-effects model (random intercept per eye; fixed effects for subgroup, time (in months from baseline), and their interaction).

During the preparation of this manuscript, the authors used Claude Opus 4.8 (Anthropic), a generative artificial-intelligence tool, to assist with language editing, formatting of the manuscript to the journal template, and the organization and drafting of selected sections of the text. The authors reviewed, edited, and verified all AI-assisted output and take full responsibility for the data, analyses, interpretations, and conclusions presented in this publication.

## 3. Results

A total of 322 patient-eye records were analyzed, including 70 treatment-naïve eyes (22%) and 252 continuation eyes (78%). The baseline clinical characteristics of the study population are summarized in Table 1.

**Figure 1:**
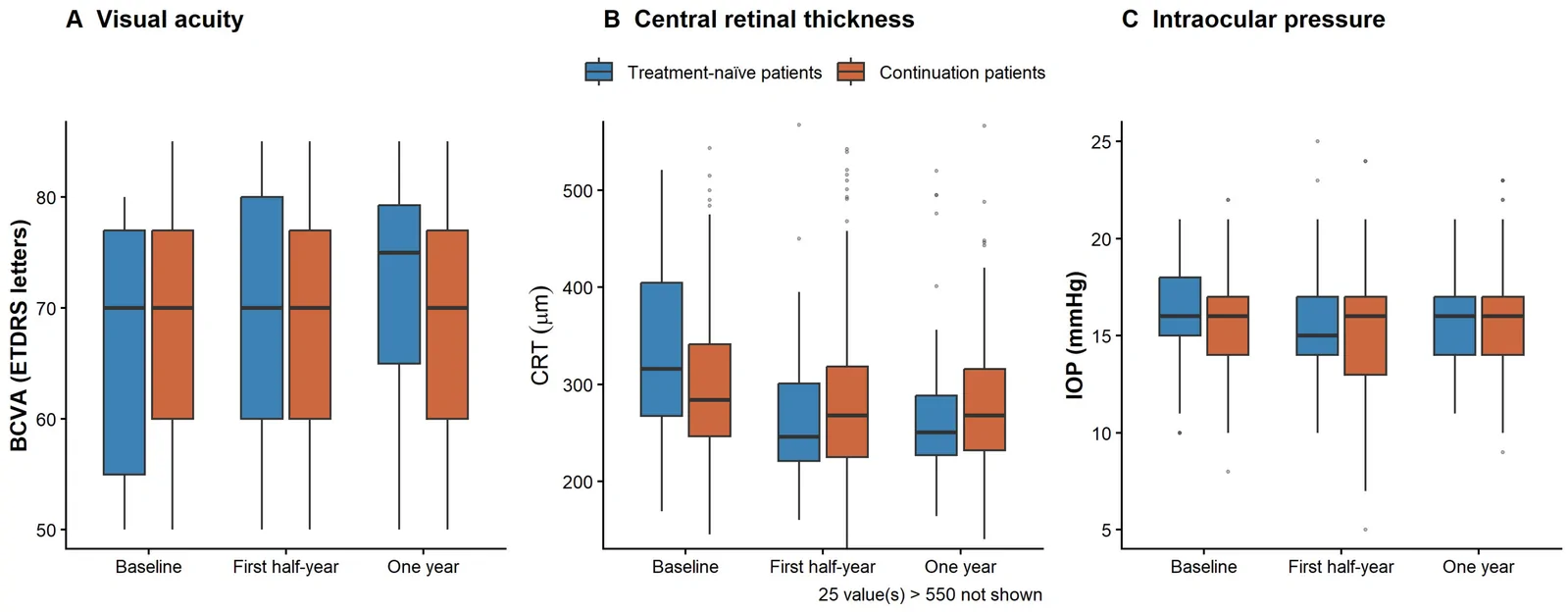
Clinical outcomes over the first year of faricimab treatment in treatment-naïve (*n* = 70) and continuation (*n* = 252) eyes. Box plots show (**A**) best-corrected visual acuity (BCVA, ETDRS letters), (**B**) central retinal thickness (CRT, µm), and (**C**) intraocular pressure (IOP, mmHg) at baseline, the first half-year, and one year. Boxes show the median and interquartile range; whiskers extend to 1.5 × IQR; points are outliers. In panel (**B**), the y-axis is truncated at 550 µm (25 values above this range not shown).

The median baseline BCVA was 70 ETDRS letters [IQR: 60, 77] and did not differ between treatment-naïve and continuation eyes (*p* = 0.59). After the first half-year, median BCVA improved versus baseline in the overall cohort and in both subgroups (all *p* ≤ 0.005), with no between-group difference (*p* = 0.68). After one year, median BCVA in the overall cohort was unchanged at 70 [60, 77] letters; the difference from baseline reached nominal significance (*p* = 0.037) but corresponded to a median change of 0 letters and is therefore not clinically meaningful. In treatment-naïve eyes, BCVA increased to 75 [66, 79] at one year (*p* < 0.001 vs. baseline), while in continuation eyes, BCVA was maintained without significant change from baseline (*p* = 0.98). Accordingly, the one-year ∆BCVA was greater in treatment-naïve than in continuation eyes (5 [0, 10] vs. 0 [−5, 5] letters; *p* < 0.001) (Figure 1A). After adjustment for baseline BCVA (ANCOVA), the treatment-naïve versus continuation difference in one-year ΔBCVA remained significant (+4.4 letters; 95% CI 2.2 to 6.5; *p* < 0.001).

Baseline central retinal thickness (CRT) for all patients was 289 [251, 354] µm, with higher values in treatment-naïve than in continuation eyes (316 [267, 405] µm vs. 284 [247, 341] µm; *p* = 0.005). CRT decreased significantly from baseline after the first half-year and remained lower at one year in all groups (all *p* ≤ 0.001). There was no significant difference in CRT between the first and second half-year within groups. Because baseline CRT differed between subgroups, between-group comparisons at follow-up were performed using changes in CRT (∆CRT). Treatment-naïve eyes demonstrated a greater reduction in CRT than continuation eyes at the first half-year (−70 [−133, −10] µm vs. −11 [−54, 15] µm; *p* < 0.001) and at one year (*p* < 0.001). There was no between-group difference in CRT from the first to the second half-year (*p* = 0.39) (Figure 1B). After adjustment for baseline CRT (ANCOVA), the greater reduction in treatment-naïve eyes was attenuated but remained significant (−29.5 µm; 95% CI −52.6 to −6.5; *p* = 0.012).

Median baseline intraocular pressure (IOP) was 16 [14, 17] mmHg and did not differ between subgroups. IOP remained stable after the first half-year in all groups. After one year, a statistically significant change was observed in the overall cohort; this was driven primarily by the continuation subgroup, in which IOP differed from baseline and between the first and second half-year (*p* = 0.028 and *p* = 0.036, respectively). Despite statistical significance, the median ∆IOP at one year remained 0 mmHg in both subgroups (below any clinically relevant threshold), but with statistically significant difference between subgroups (treatment-naïve: 0 [−2, 1] mmHg vs. continuation: 0 [−1, 2] mmHg; *p* = 0.048) (Figure 1C).

**Figure 2:**
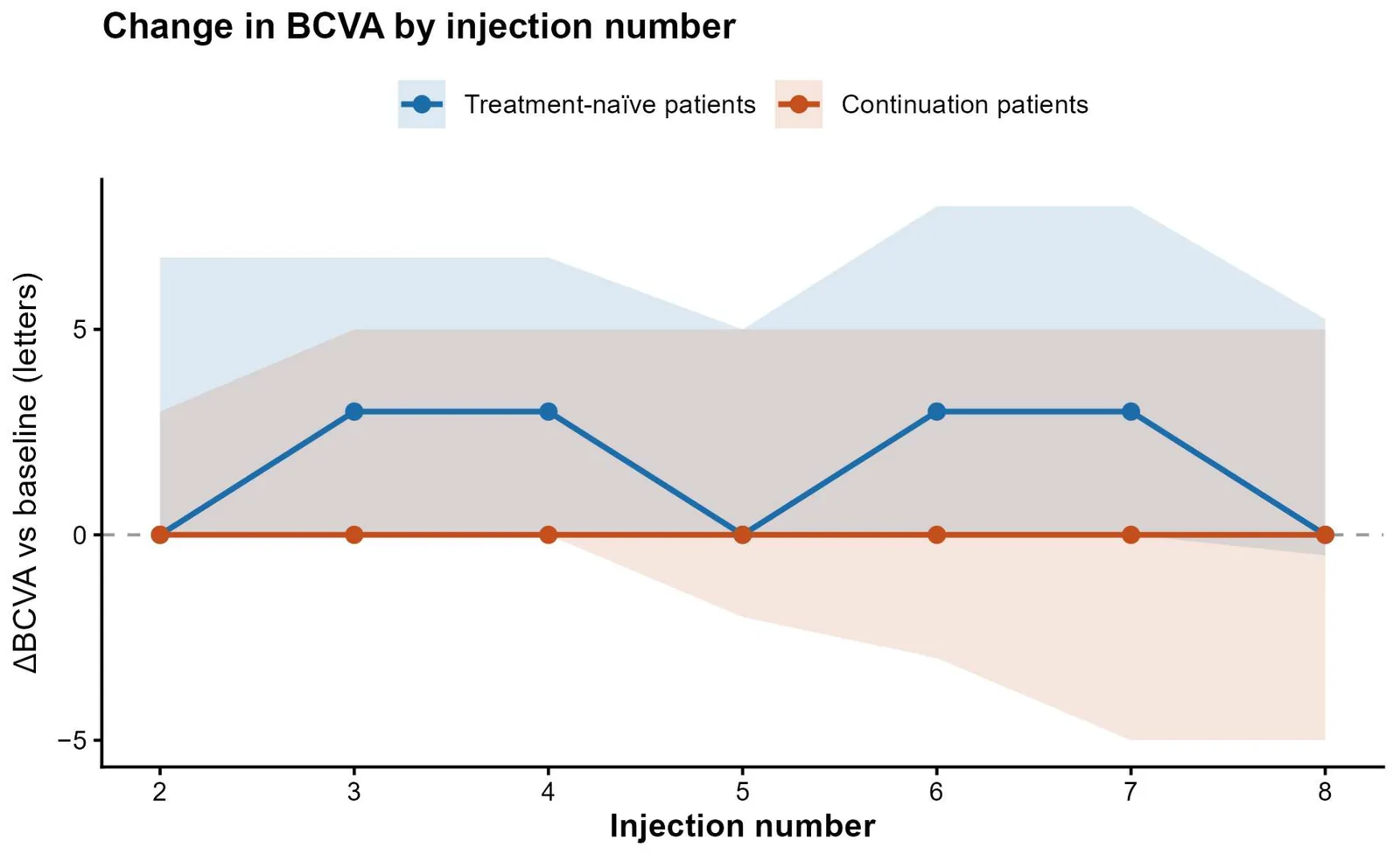
Change in BCVA relative to baseline (first faricimab injection) by injection number. Lines show the median change (ETDRS letters); shaded bands denote the interquartile range. The dashed line marks no change from baseline. Injection 1 corresponds to baseline (change of zero by definition); the x-axis therefore starts at injection 2.

Changes in BCVA according to injection number are presented in Supplementary Table S1 and Figure 2. At baseline (injection 1), the median BCVA in the overall cohort was 70 [60–77] ETDRS letters and did not differ between treatment-naïve and continuation eyes (*p* > 0.05).

Across consecutive injections, median BCVA remained stable in the overall cohort, with a modest improvement observed mainly in treatment-naïve eyes during the early treatment phase, whereas continuation eyes were maintained. In a linear mixed-effects model of BCVA over time (random intercept per eye), the treatment-naïve and continuation trajectories diverged (group × time interaction +0.35 letters/month; 95% CI 0.22 to 0.48; *p* < 0.001), corresponding to a treatment-naïve advantage of approximately 3.5 letters at one year and consistent with the baseline-adjusted analysis.

**Figure 3:**
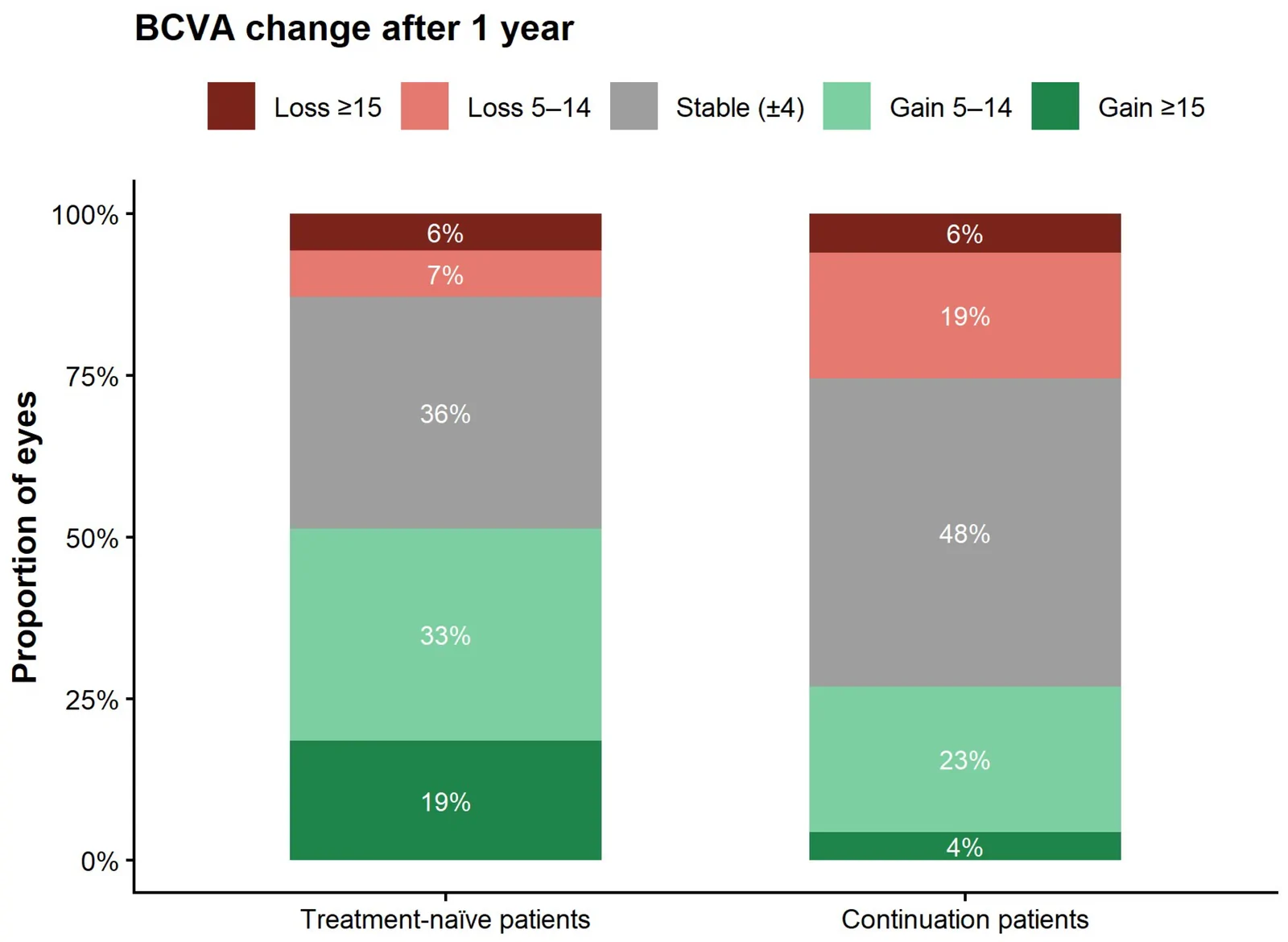
Distribution of one-year BCVA change by standard ETDRS categories (loss ≥ 15, loss 5–14, stable ±4, gain 5–14, gain ≥ 15 letters) in treatment-naïve and continuation eyes. Bars show the proportion of eyes in each category. Percentages may not sum to 100 owing to rounding.

After one year of treatment, 50/322 (16%) patients achieved a clinically meaningful improvement in BCVA (≥10-letter gain), while 42/322 (13%) experienced a clinically meaningful deterioration (≥10-letter loss). The proportion with ≥10-letter improvement was higher in the treatment-naïve than in the continuation subgroup (0.27 vs. 0.12; *p* = 0.004; 95% CI for the difference, 0.03–0.27), whereas the proportion with ≥10-letter deterioration did not differ between subgroups (*p* = 0.29) (Table 2). The distribution of one-year BCVA change by standard ETDRS categories is shown in Figure 3: among treatment-naïve eyes, 52% gained ≥5 letters (33% gained 5–14 and 19% gained ≥15) and 36% remained stable (±4 letters), whereas continuation eyes were predominantly stable (48%), with 27% gaining ≥5 letters and 25% losing ≥5 letters.

**Figure 4:**
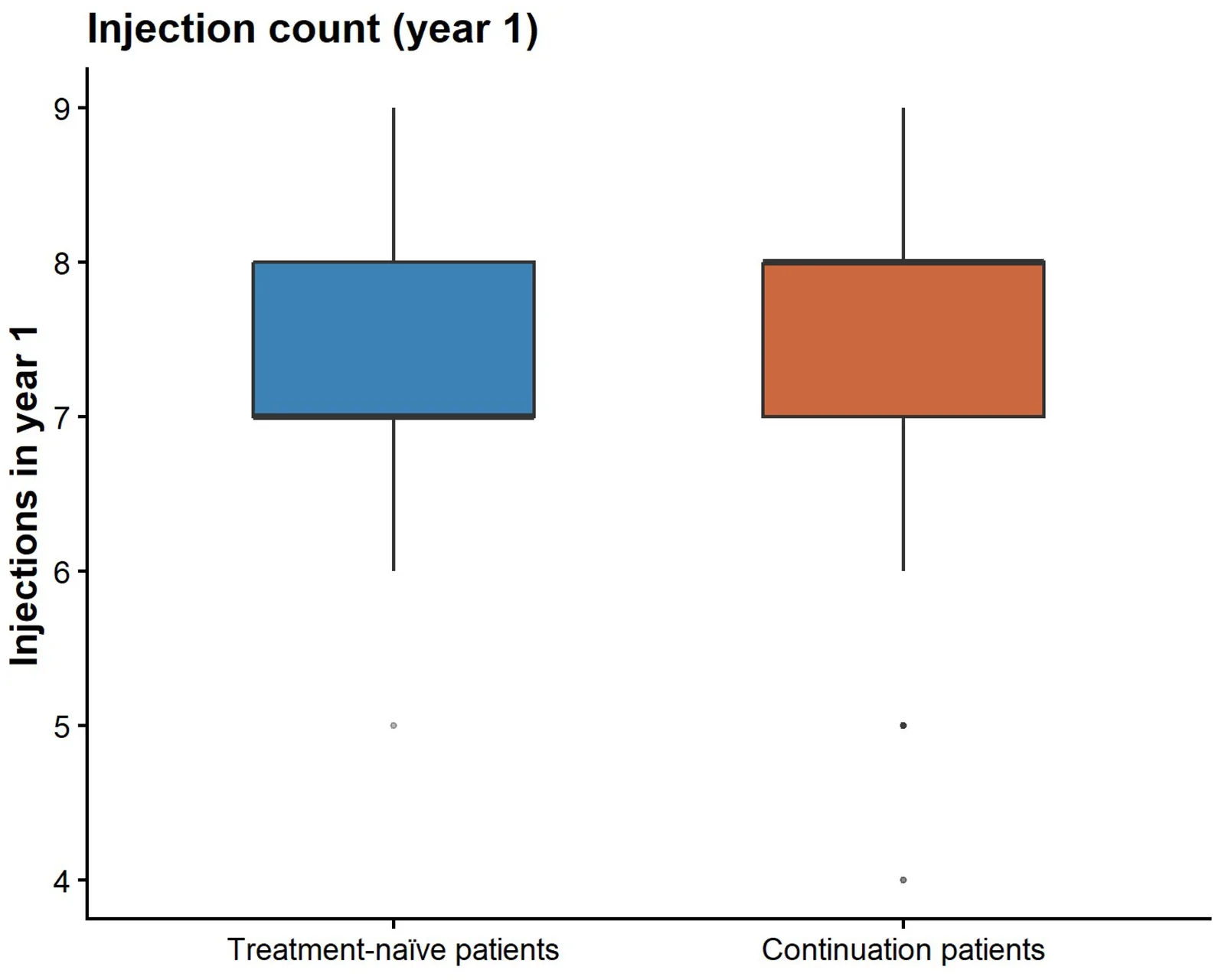
Number of faricimab injections during the first year of treatment in treatment-naïve (*n* = 70) and continuation (*n* = 252) eyes. Boxes show the median and interquartile range; whiskers extend to 1.5 × IQR; points are outliers. Blue, treatment-naïve eyes; orange, continuation eyes.

During the first year, patients received a median of 8 injections [IQR: 7, 8], fewer in treatment-naïve than in continuation eyes; injection numbers fell from the first to the second half-year in all groups. Full counts and between-group comparisons are given in Table 1 and Figure 4.

**Figure 5:**
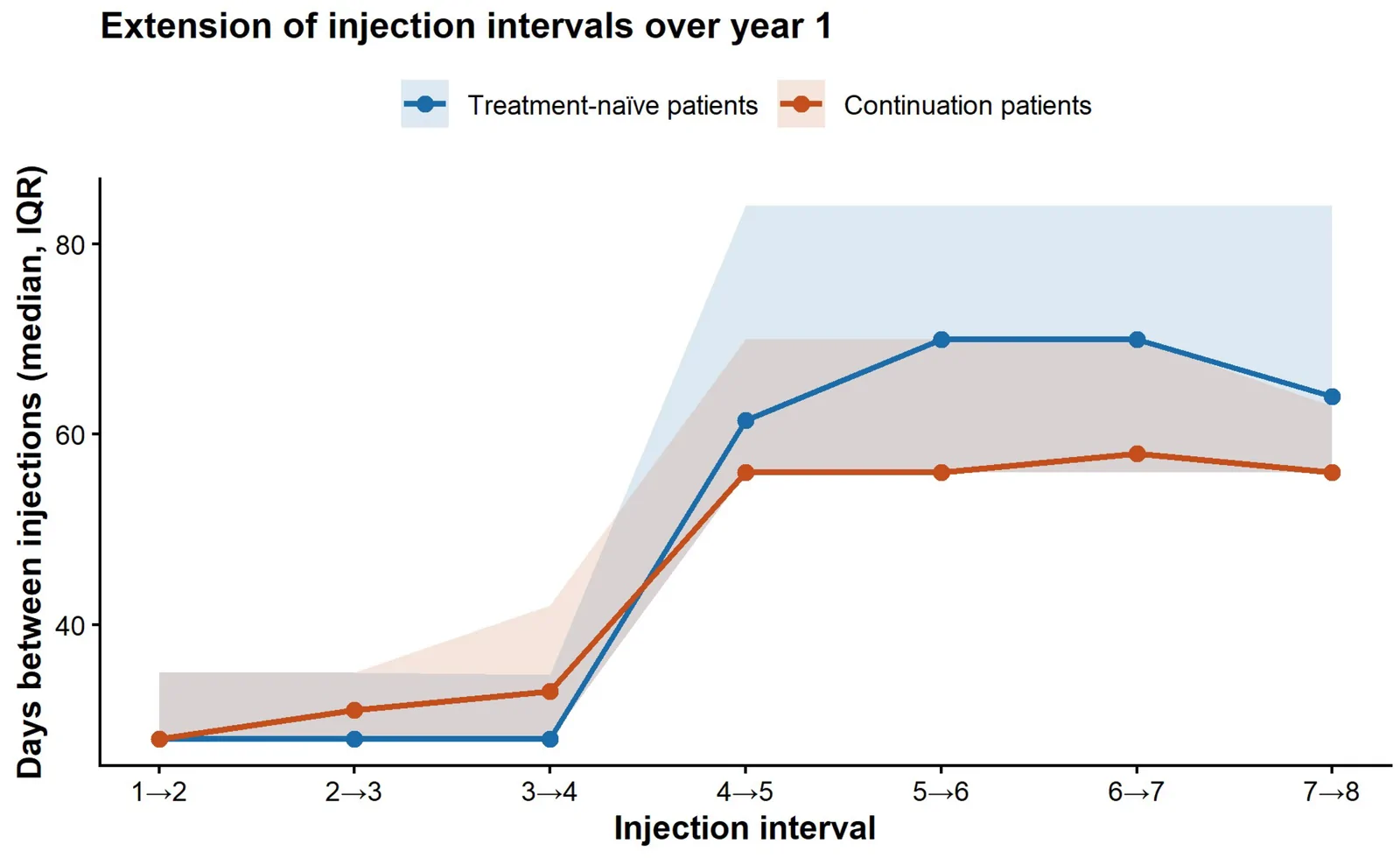
Extension of injection intervals over the first year. Lines show the median number of days between consecutive faricimab injections in treatment-naïve and continuation eyes; shaded bands denote the interquartile range (intervals 1 → 2 through 7 → 8).

Injection intervals during the first year of treatment are presented in Supplementary Table S3 and Figure 5. The shortest intervals were observed at the beginning of treatment, followed by gradual extension over time.

In the overall cohort, the only substantial change in interval was the step from the loading phase to maintenance: the median interval rose from 29 [28–36] days between the third and fourth injections to 56 [56–75] days between the fourth and fifth (*p* < 0.001), whereas other adjacent intervals differed by ≤4 days.

The pattern was consistent in both subgroups. Treatment-naïve patients demonstrated slightly longer intervals in the later phases (e.g., 62 [56–70] days) compared with continuation patients (56 [49–63] days).

Overall, injection intervals remained close to four weeks in the initial phase, followed by a marked extension to approximately eight weeks in the later phase of the first year.

No significant differences in BCVA were observed between treatment-naïve and continuation eyes at corresponding injection visits (all *p* > 0.05).

Changes in BCVA (∆BCVA) relative to baseline and to the previous injection are summarized in Supplementary Table S2. Compared with baseline (injection 1), statistically significant improvements were observed after selected injections in the overall cohort and in the treatment-naïve subgroup (*p* < 0.05 for the significant comparisons; Supplementary Table S1), while changes in the continuation subgroup were small.

## 4. Discussion

In this real-world analysis of 322 eyes treated with faricimab within the Polish national drug program, one year of therapy was associated with a modest functional improvement in treatment-naïve eyes and maintenance of vision in eyes switched from other anti-VEGF agents, together with a significant anatomical improvement across all subgroups and a decline in injection frequency and extension of treatment intervals over follow-up after the mandated loading phase. To our knowledge, this is among the first analyses of faricimab within the Polish Therapeutic Program Monitoring System, adding national real-world evidence to a rapidly growing international literature [7].

Real-world outcomes of anti-VEGF therapy frequently fall short of trial results. In routine practice, patients often receive fewer injections and less regular follow-up, which can translate into inferior functional outcomes; multi-country real-life data have consistently shown that visual acuity gains in clinical practice are smaller than those reported in randomized trials, largely reflecting undertreatment and variability in treatment regimens [12]. Large observational registries and national databases have therefore become essential tools for evaluating treatment effectiveness in routine care, providing the context in which the present SMPT-based analysis should be interpreted.

Evidence on faricimab is now expanding beyond the trial setting. A single-arm systematic review and meta-analysis pooling 21 studies (1864 eyes) reported significant improvements in visual acuity and central macular thickness after faricimab in nAMD across both randomized and real-world cohorts [13], while the largest real-world analysis to date (FARETINA-AMD; approximately 29,000 eyes) reported visual gains in treatment-naïve eyes and maintenance of vision in previously treated eyes [14]. We interpret our own results against this expanding evidence base below. Direct head-to-head data against the most durable current regimens remain limited. A network meta-analysis of aflibercept 8 mg versus faricimab treat-and-extend reported no significant difference in visual acuity or central retinal thickness over one to two years, with a somewhat lower projected injection frequency for aflibercept 8 mg; the analysis was, however, industry-sponsored and based on indirect comparison [15]. A protocol-standardised in silico comparison similarly estimated closely matched one-year injection frequencies for faricimab 6 mg and aflibercept 8 mg (approximately seven injections each), indicating that apparent between-agent differences in durability may partly reflect differences in trial regimens rather than intrinsic pharmacology [16]. Our single-arm data cannot adjudicate between agents, but the interval extension and injection counts we observed are consistent with the faricimab estimates in these analyses.

The lower maintenance-phase injection frequency observed here may have implications for the Polish healthcare system. Treatment of nAMD is the most populous of the national drug programs [17], and its capacity is constrained by the number of injection and monitoring visits that ophthalmology services can deliver [8]. Modelling by Seweryn et al. estimated that, by reducing injection frequency by approximately 15%, faricimab could free capacity to treat several thousand additional patients within the program without additional resources [17]. Our real-world data are compatible with the interval assumptions used in such projections; our single-arm, persister-cohort data cannot themselves validate that model, but they indicate that maintenance intervals of approximately 8 weeks are attainable in Polish routine practice and reinforce the potential of longer-acting agents to help meet the growing demand for retinal disease treatment in an ageing population.

Our functional findings are consistent with the pivotal TENAYA/LUCERNE trials [4] and with the largest real-world cohort reported to date, FARETINA-AMD, in which treatment-naïve eyes gained a mean of 2.0 letters, while previously treated eyes maintained vision over one year [14]. The median 5-letter gain we observed in treatment-naïve eyes lies within this range and is concordant with other treatment-naïve real-world series reporting early-to-one-year visual improvement (e.g., FARI-LATAM, +9.1 letters at a mean of ~26 weeks [18]; loading-phase cohorts [19]; a one-year Japanese cohort [20]). Notably, our treatment-naïve eyes had a relatively high median baseline BCVA (70 letters) compared with the treatment-naïve populations of TENAYA/LUCERNE (mean ≈59–61 letters [4]) and FARETINA-AMD (≈57 letters [21]), leaving less headroom for improvement and contributing to the modest median gain (ceiling effect). The differential response between subgroups—a greater median gain and a higher proportion of clinically meaningful (≥10-letter) improvement in treatment-naïve than in continuation eyes (27% vs. 12%)—is expected. Continuation eyes entered faricimab therapy with already-preserved acuity (median 70 letters) after prior anti-VEGF treatment, leaving limited room for further gains, the therapeutic goal here being disease stabilization rather than improvement. Maintenance of vision in our continuation eyes compares favourably with switch series in more heavily pretreated populations; Janmohamed et al., for instance, reported a small but significant BCVA decline (median 70 → 62 letters) at 12 months in eyes with a median of 18 prior injections [22], underscoring that visual outcomes after switching depend strongly on the extent of prior treatment and disease chronicity. Early real-world reports (TRUCKEE [23]; Stanga et al. [24]) and the pooled single-arm meta-analysis of 1864 eyes [13] likewise support visual improvement or stability with faricimab, although the small samples and short follow-up of the earliest series warrant caution.

Anatomically, CRT decreased significantly in all subgroups, with a greater reduction in treatment-naïve eyes—a pattern driven by their higher baseline CRT and greater residual exudative activity. This mirrors FARETINA-AMD (CST reduction of −53.1 µm in naïve vs. −28.5 µm in previously treated eyes [14], with earlier data in Ali et al. [21]) and switch cohorts in which faricimab produced robust anatomical improvement even when visual acuity remained stable (Wolfrum et al., CRT 335.8→260.0 µm [25]; Bleidissel et al., significant reductions in intraretinal and subretinal fluid and central subfield thickness [26]; Xu et al., CMT 447.7 → 327.3 µm in refractory eyes [27]; Han et al., high rates of fluid resolution after three loading injections [19]). The dissociation seen in our continuation eyes—a modest anatomical improvement, with substantial variability, despite essentially unchanged acuity—is a recurring theme in the switch literature and likely reflects both the ceiling effect and irreversible structural damage (fibrosis, atrophy) accrued during long-standing disease, which limits functional recovery despite improved fluid control. Dual inhibition of VEGF-A and angiopoietin-2, the latter promoting vascular stabilization and reducing inflammation, provides a mechanistic rationale for the anatomical benefit observed after switching [4]. Anatomical improvement does not necessarily translate into proportional visual gains, because irreversible retinal damage may already be present, particularly in previously treated eyes. Emerging intraocular cytokine data suggest that pathways beyond VEGF contribute to faricimab’s durability [20].

A central observation was the extension of dosing intervals after the loading phase. Injection frequency fell from a median of 5 injections in the first half-year to 3 in the second, and inter-injection intervals extended from approximately four weeks during the loading phase to approximately eight weeks (the programme minimum maintenance interval) later in the first year. This is concordant with FARETINA-AMD, where mean injections decreased from 4.0–4.2 to 2.4–3.2 between the first and second six-month periods [14], and with switch cohorts reporting a doubling of intervals (Janmohamed et al., 4→8 weeks [22]; Bleidissel et al., 5 → 8 weeks, with one-third of eyes reaching ≥10 weeks [26]) and international series with last mean intervals of ~9–12 weeks (FARI-LATAM [18]). Notably, treatment-naïve eyes required fewer total injections and achieved longer late-phase intervals than continuation eyes, although this one-injection difference, with overlapping interquartile ranges, should not be over-interpreted as faster disease stabilization. Intervals in our cohort nonetheless remained shorter than the up-to-16-week maximum reported in the pivotal trials [4], which probably reflects the conservative, protocol-driven extension typical of routine practice and the specific administrative and monitoring requirements of the B.70 program—echoing the well-documented gap between trial and real-world dosing [12]. Whether a structured four-dose induction (“re-loading”) at switch, rather than immediate treat-and-extend, would enable longer intervals, as suggested by Menghini et al. [28], merits evaluation within the Polish program.

Consistent with the established profile of faricimab, intraocular pressure remained clinically stable throughout follow-up, with no clinically meaningful change in either subgroup (median ∆IOP of 0 mmHg). Although adverse events, intraocular inflammation, and reasons for discontinuation are not recorded in the SMPT dataset and could not be assessed here, the broader real-world literature reports a low incidence of treatable intraocular inflammation and only rare serious events [13,23].

This study has several strengths, including the use of nationwide, prospectively collected registry data reflecting unselected routine practice, a clinically relevant minimum follow-up of 360 days, and the analysis of both treatment-naïve and switched patients within a single reimbursement framework. Several limitations should be acknowledged. First, the observational, non-randomized, single-arm design without a concurrent comparator precludes conclusions about comparative effectiveness relative to other anti-VEGF agents; the results should be interpreted as associations. Second, analyses were performed on complete-case data at each time point without imputation, and the number of eyes with available data decreased at later injections, which may introduce attrition bias. Third, BCVA was recorded in Snellen notation during routine care and converted to approximate ETDRS letters [11]; although this is a validated and intuitive approach, it does not remove the imprecision inherent to non-protocol Snellen measurement, which therefore remains a limitation. Because Snellen-derived ETDRS scores are discrete and tied to whole Snellen lines, a median change of 5 letters corresponds to a single line and lies at the resolution limit of the measurement; we therefore gave primacy to the ≥10-letter threshold analysis. The registry also records retinal thickness only as a single integer value without specifying the measurement type (e.g., central subfield thickness), the OCT device, or the acquisition protocol [29], so anatomical values are device-heterogeneous and investigator-entered; individual-level demographics (age, sex) and lens status were likewise not part of the analysed extract, although the demographic profile of the Polish nAMD programme population (mean age ~75 years, ~63% female) has been described previously [7]. Because laterality and second-eye linkage were unavailable, residual non-independence related to bilateral treatment cannot be entirely excluded. Fourth, the SMPT dataset does not capture detailed OCT morphology (e.g., intraretinal versus subretinal fluid, pigment epithelial detachment, macular neovascularization subtype), fibrosis, or the specific reasons for switching, limiting mechanistic interpretation and direct comparison with studies stratified by fluid compartment [19,26]. Fifth, because we performed multiple unadjusted comparisons, some statistically significant results—particularly those with median changes at or near zero, such as the change in intraocular pressure—likely reflect the large sample size rather than a clinically relevant effect and should not be over-interpreted; throughout, we prioritised the magnitude of change and the proportion of eyes reaching predefined clinical thresholds over nominal *p*-values. The covariance and mixed-effects analyses further assume that missing follow-up data are missing at random and approximate the loading-to-maintenance course with a linear time term; both should therefore be regarded as supportive rather than confirmatory. Finally, follow-up was limited to one year; longer-term data are needed to confirm the durability of visual and anatomical benefits and the sustainability of extended dosing intervals within the program.

By design, eligibility required participation in the program for at least 360 days; patients who left faricimab treatment earlier were therefore not captured. Because the SMPT dataset does not record the reason for discontinuation, we could not distinguish protocol-defined program completion from switching to another agent, loss to follow-up, an adverse event, or death, nor separate planned exit from discontinuation due to inadequate response or intolerance. The analysed cohort consequently represents patients who persisted on faricimab for at least one year, and the reported outcomes may overestimate effectiveness and treatment durability relative to all patients who initiated faricimab. The present study should therefore be interpreted as an effectiveness analysis among patients who persisted on faricimab for at least one year rather than an intention-to-treat analysis of all patients initiating therapy. Because the data extend only to April 2025, the ≥360-day requirement means that, in practice, only patients initiating faricimab before approximately April 2024 could qualify. Conditioning on persistence is expected to bias the observed injection intervals toward shorter values, the injection counts upward, and the visual outcomes toward responders.

In addition, the continuation subgroup was heterogeneous with respect to prior therapy. The SMPT dataset does not capture the number or duration of previous anti-VEGF injections, the specific agents used, disease chronicity, or the reason for switching to faricimab. As visual and anatomical outcomes after switching depend strongly on prior treatment burden and disease duration [22], the maintenance of vision observed in this subgroup should be interpreted cautiously; it may encompass eyes switched early in their disease course as well as long-standing, heavily pretreated eyes with limited functional reserve. This heterogeneity also limits direct interpretation of the treatment-naïve versus continuation contrast.

## 5. Conclusions

In this real-world analysis of the Polish national drug program, one year of faricimab therapy was associated with stable visual acuity and significant anatomical improvement in patients with neovascular age-related macular degeneration. Visual outcomes were maintained in previously treated eyes and modestly improved in treatment-naïve eyes (median one-year gain of 5 ETDRS letters), while central retinal thickness decreased significantly in all subgroups. Injection intervals extended progressively during follow-up, with fewer injections in the second than in the first half-year, reflecting extension of dosing intervals after the loading phase, without loss of functional benefit. These findings are consistent with pivotal trial data and with large international real-world cohorts. Given the observational, single-arm, persister-only design, they should be interpreted as associations rather than as evidence of comparative effectiveness.

## Figures and Tables

**Table 1:** Baseline characteristics and 1-year clinical outcomes of patients treated with faricimab within the national drug program.

Variable	Treatment Duration	All Patients	Treatment- Naïve Patients	Continuation Patients	*p*-Value
Number of eyes (patients)	First year of treatment	322	70	252	
Number of injections (median (IQR))	First half-year	5 (4, 5)	5 (5, 5)	5 (4, 5)	0.07
	Second half-year	3 (2, 3)	2 (2, 3)	3 (3, 3)	<0.001
	*p*-value (first half-year vs second half-year)	<0.001	<0.001	<0.001	
	First year of treatment	8 (7, 8)	7 (7, 8)	8 (7, 8)	0.014
BCVA, ETDRS letter score (median (IQR))	Baseline	70 (60, 77)	70 (55, 77)	70 (60, 77)	0.59
	First half-year	70 (60, 77)	70 (60, 80)	70 (60, 77)	0.68
	*p*-value (first half-year vs. baseline)	<0.001	0.005	0.004	
	One year	70 (60, 77)	75 (66, 79)	70 (60, 77)	0.023
	*p*-value (first year vs. baseline)	0.037	<0.001	0.98	
	*p*-value (first half-year vs. second half-year)	0.016	0.055	<0.001	
∆ BCVA, ETDRS letter score (median (IQR))	First half-year vs. baseline	0 (0, 5)	0 (0, 5)	0 (−2, 5)	0.12
	First year vs. baseline	0 (−2.75, 5)	5 (0, 10)	0 (−5, 5)	<0.001
	First half-year vs. second half-year	0 (−5, 5)	0 (0, 5)	0 (−5, 0)	<0.001
Intraocular pressure, mmHg (median (IQR))	Baseline	16 (14, 17)	16 (15, 18)	16 (14, 17)	0.33
	First half-year	16 (13, 17)	15 (14, 17)	16 (13, 17)	0.51
	*p*-value (first half-year vs. baseline)	0.89	0.16	0.53	
	One year	16 (14, 17)	16 (14, 17)	16 (14, 17)	0.32
	*p*-value (first year vs. baseline)	0.13	0.31	0.028	
	*p*-value (first half-year vs. second half-year)	0.032	0.48	0.036	
∆ Intraocular pressure, mmHg (median (IQR))	First half-year vs. baseline	0 (−2, 2)	0 (−2.75, 1)	0 (−1.25, 2)	0.09
	First year vs. baseline	0 (−1, 2)	0 (−2, 1)	0 (−1, 2)	0.048
	First half-year vs. second half-year	0 (−1, 2)	0 (−1, 1)	0 (−1, 2)	0.76
Retinal thickness, µm (median (IQR))	Baseline	289 (251, 354)	316 (267, 405)	284 (247, 341)	0.005
	First half-year	265 (223, 314)	246 (221, 301)	268 (225, 318)	0.103
	*p*-value (first half-year vs. baseline)	<0.001	<0.001	<0.001	
	One year	265 (231, 311)	251 (227, 288)	268 (232, 316)	0.087
	*p*-value (first year vs. baseline)	<0.001	<0.001	0.001	
	*p*-value (first half-year vs. second half-year)	0.78	0.39	0.51	
∆ Retinal thickness, µm (median (IQR))	First half-year vs. baseline	−16 (−71, 10)	−70 (−133, −10)	−11 (−54, 15)	<0.001
	First year vs. baseline	−20 (−69, 20)	−53 (−130, −8)	−10 (−62, 27)	<0.001
	First half-year vs. second half-year	−1 (−30, 33)	5 (−20, 28)	−2 (−38, 38)	0.39

Within-group comparisons were made with the Wilcoxon signed-rank test, which assesses the symmetry of the distribution of paired differences (its estimator is the Hodges–Lehmann pseudomedian) rather than the sample median; a statistically significant *p*-value can therefore arise even when the median change is 0. Throughout, Δ denotes the later minus the earlier value; a positive Δ indicates an improvement in BCVA, and a negative Δ indicates a reduction in retinal thickness.

**Table 2:** Patients with clinically meaningful improvement/deterioration in BCVA after one year of treatment.

Variable	All Patients		Treatment-Naïve Patients		Continuation Patients			
	Number of Patients	Proportion	Number of Patients	Proportion	Number of Patients	Proportion	*p*-Value **	95% CI
clinically meaningful improvement in BCVA	50	0.16	19	0.27	31	0.12	0.004	(0.03, 0.27)
clinically meaningful deterioration in BCVA	42	0.13	6	0.09	36	0.14	0.29	(−0.14, 0.02)

** *p*-values are from the two-sided chi-square test; 95% confidence intervals refer to the between-group difference in proportions (treatment-naïve minus continuation, Wald method). Proportions denote the fraction of eyes with a ≥10-letter change at one year.

## Data Availability

The data analyzed in this study were obtained from the Therapeutic Program Monitoring System (SMPT), operated by the Polish National Health Fund (NFZ). Restrictions apply to the availability of these data, which were used under authorization for the present study and are not publicly available. Access to the data is subject to the permission of the NFZ.

## Supplementary Materials

The following supporting information can be downloaded at: https://www.mdpi.com/article/10.3390/jcm15197602/s1. Table S1: BCVA (ETDRS letters, median [IQR]) at baseline and subsequent injections; Table S2: Change in BCVA (ETDRS letters, median [IQR]) relative to baseline and to the previous injection; Table S3: Comparison of injection intervals (days, median [IQR]) over the first year of treatment. Per-injection patient counts in Tables S1–S3 decrease at later injections because follow-up length varied, and therefore need not equal the one-year totals in Table 1.
