# Supplementary material for: Effectiveness and Treatment Burden of Faricimab in Neovascular AMD: A Real-World Analysis of Treatment-Naïve and Previously Treated Patients in Poland
Source: J Clin Med. 2026 Sep 30;15(19):7602. doi: 10.3390/jcm15197602 (PMC13665884; doi:10.3390/jcm15197602)
Supplement: Supplementary file 1 [file jcm-15-07602-s001.zip › jcm-4531131-supplementary.pdf]

MDPI

Journal of Clinical Medicine

Supplementary Materials

Effectiveness and Treatment Burden of Faricimab in Neovascular AMD: A Real-World Analysis of Treatment-Naïve and Previously Treated Patients in Poland.

Supplementary Tables S1–S3 present per-injection outcomes summarised graphically in the main text (Figures 2 and 5). All p-values are unadjusted and reported descriptively.

Supplementary Table S1. BCVA (ETDRS letters, median [IQR]) at baseline and subsequent injections

p-value**	Continuation patients		Treatment-naïve patients		All patients		Injection number	Variable
	Parameter value	Number of patients	Parameter value	Number of patients	Parameter value	Number of patients		
0.59	70 (60, 77)	252	70 (55, 77)	70	70 (60, 77)	322	Injection 1 (baseline)	
0.82	70 (60, 77)	252	70 (61, 77)	70	70 (60, 77)	322	Injection 2	
	<0.05		<0.05		<0.05		p-value (Injection 2 vs Injection 1)	
0.95	70 (60, 77)	252	70 (60, 77)	70	70 (60, 77)	322	Injection 3	
	<0.05		<0.05		<0.05		p-value (Injection 3 vs njection 1)	
	<0.05		0.32		<0.05		p-value (Injection 3 vs Injection 2)	
0.86	70 (60, 77)	252	70 (65, 77)	70	70 (60, 77)	322	Injection 4	
	<0.05		<0.05		<0.05		p-value (Injection 4 vs Injection 1)	
	0.85		0.92		0.83		p-value (Injection 4 vs Injection 3)	
0.36	70 (60, 77)	252	70 (60, 79)	70	70 (60, 77)	322	Injection 5	
	0.15		<0.05		<0.05		p-value (Injection 5 vs Injection 1)	BCVA, ETDRS letter score (median [IQR]) – by number of injections
	<0.05		0.96		<0.05		p-value (Injection 5 vs Injection 4)	
0.16	70 (60, 77)	248	73 (61, 77)	70	70 (60, 77)	318	Injection 6	
	0.10		<0.05		<0.05		p-value (Injection 6 vs Injection 1)	
	0.88		0.27		0.49		p-value (Injection 6 vs Injection 5)	
0.10	70 (60, 77)	241	75 (60, 77)	69	70 (60, 77)	310	Injection 7	
	0.38		<0.05		<0.05		p-value (Injection 7 vs Injection 1)	
	0.90		0.94		0.13		p-value (Injection 7 vs Injection 6)	
0.12	70 (60, 77)	204	70 (60, 77)	49	70 (60, 77)	253	Injection 8	
	0.99		<0.05		0.14		p-value (Injection 8 vs Injection 1)	
	0.61		0.76		0.52		p-value (Injection 8 vs Injection 7)	

Within-group comparisons were made with the Wilcoxon signed-rank test, which assesses the symmetry of the distribution of paired differences (its estimator is the Hodges–Lehmann pseudomedian) rather than the sample median; a statistically significant p-value can therefore arise even when the median change is 0. All p-values are unadjusted and reported descriptively.

Supplementary Table S2. Change in BCVA (ETDRS letters, median [IQR]) relative to baseline and to the previous injection

p-value**	Continuation patients		Treatment-naïve patients		All patients		Injection number	Variable
	Parameter value	Number of patients	Parameter value	Number of patients	Parameter value	Number of patients		
<0.05	0 (0, 3)	252 vs 252	0 (0, 6.75)	70 vs 70	0 (0, 5)	322 vs 322	Injection 2 vs Injection 1	BCVA, ETDRS letter score (median [IQR]) – Relative to Injection 1 (baseline)
<0.05	0 (0, 5)	252 vs 252	3 (0, 6.75)	70 vs 70	0 (0, 5)	322 vs 322	Injection 3 vs Injection 1	
0.14	0 (0, 5)	252 vs 252	3 (0, 6.75)	70 vs 70	0 (0, 5)	322 vs 322	Injection 4 vs Injection 1	
<0.05	0 (-3, 5)	252 vs 252	0 (0, 5)	70 vs 70	0 (-2, 5)	322 vs 322	Injection 5 vs Injection 1	
<0.05	0 (-3, 5)	248 vs 252	3 (0, 7.75)	70 vs 70	0 (-2, 5)	318 vs 322	Injection 6 vs Injection 1	
<0.05	0 (-5, 5)	241 vs 252	3 (0, 10)	69 vs 70	0 (-3, 5)	310 vs 322	Injection 7 vs Injection 1	
<0.05	0 (-5, 5)	204 vs 252	3 (0, 8)	49 vs 70	0 (-3, 5)	253 vs 322	Injection 8 vs Injection 1	
0.71	0 (0, 2)	252 vs 252	0 (0, 3)	70 vs 70	0 (0, 2.75)	322 vs 322	Injection 3 vs Injection 2	BCVA, ETDRS letter score (median [IQR]) – Relative to previous injection
0.89	0 (-0.5, 0)	252 vs 252	0 (-2, 0)	70 vs 70	0 (-2, 0)	322 vs 322	Injection 4 vs Injection 3	
<0.05	0 (-5, 0)	252 vs 252	0 (0, 2.75)	70 vs 70	0 (-5, 0)	322 vs 322	Injection 5 vs Injection 4	
0.21	0 (-0.5, 2)	248 vs 252	0 (0, 3)	70 vs 70	0 (0, 2.75)	318 vs 322	Injection 6 vs Injection 5	
0.99	0 (-2, 0)	241 vs 248	0 (-2, 0)	69 vs 70	0 (-2, 0)	310 vs 318	Injection 7 vs Injection 6	
0.92	0 (0, 0)	204 vs 241	0 (-2, 3)	49 vs 69	0 (0, 0)	253 vs 310	Injection 8 vs Injection 7	

Supplementary Table S3. Comparison of injection intervals (days, median [IQR]) over the first year of treatment

p-value**	Continuation patients		Treatment-naïve patients		All patients		Interval number: injection numbers	Variable
	Parameter value	Number of patients	Parameter value	Number of patients	Parameter value	Number of patients		
0.17	28 (28, 35)	252 to 252	28 (28, 35)	70 to 70	28 (28, 35)	322 to 322	interval 1: Injection 1 to Injection 2	Injection intervals (days), median [IQR]
0.20	31 (28, 35)	252 to 252	28 (28, 35)	70 to 70	30 (28, 35)	322 to 322	interval 2: Injection 2 to Injection 3	
	0.30		0.30		0.17		p-value (interval 1 vs interval 2)	
<0.05	33 (28, 42)	252 to 252	28 (28, 34.75)	70 to 70	29 (28, 35.75)	322 to 322	interval 3: Injection 3 to Injection 4	
	<0.05		1		<0.05		p-value (interval 2 vs interval 3)	
0.29	56 (56, 70)	252 to 252	61.5 (56, 84)	70 to 70	56 (56, 75.25)	322 to 322	interval 4: Injection 4 to Injection 5	
	<0.05		<0.05		<0.05		p-value (interval 3 vs interval 4)	
<0.05	56 (56, 70)	252 to 248	70 (56, 84)	70 to 70	56 (56, 77)	322 to 318	interval 5: Injection 5	

							to Injection 6
	0.45		0.07		0.75		p-value (interval 4 vs interval 5)
<0.05	58 (56, 70)	248 to 241	70 (56, 84)	70 to 69	60.5 (56, 77)	318 to 310	interval 6: Injection 6 to Injection 7
	<0.05		0.39		<0.05		p-value (interval 5 vs interval 6)
<0.05	56 (56, 63)	241 to 204	64 (56, 84)	69 to 49	56 (56, 70)	310 to 253	interval 7: Injection 7 to Injection 8
	0.30		0.13		0.90		p-value (interval 6 vs interval 7)

Within-group comparisons were made with the Wilcoxon signed-rank test, which assesses the symmetry of the distribution of paired differences (its estimator is the Hodges–Lehmann pseudomedian) rather than the sample median; a statistically significant p-value can therefore arise even when the median change is 0. All p-values are unadjusted and reported descriptively.
